# Caffeine Consumption and Depression, Anxiety, and Stress Levels Among University Students in Medina: A Cross-Sectional Study

**DOI:** 10.7759/cureus.48018

**Published:** 2023-10-31

**Authors:** Nadir M Makki, Shouq T Alharbi, Abdulrahman M Alharbi, Ahad S Alsharif, Ahmed M Aljabri

**Affiliations:** 1 Psychiatry, Taibah University, Medina, SAU; 2 College of Medicine, Taibah University, Medina, SAU

**Keywords:** taibah university, coffee, caffeine, student, stress, anxiety, depression

## Abstract

Introduction

Caffeine is a psychoactive stimulant frequently found in coffee, tea, energy drinks, and some medications. Various mental health challenges, including stress, anxiety, and depression, commonly affect college students. Moreover, an individual's mental and physical health can be significantly impacted by stress, anxiety, and depression. However, the impact of caffeine on mental health, particularly its association with depressive and anxiety symptoms, remains inconclusive. Thus, this study aimed to evaluate the amount of caffeine consumed by university students and its association with depression, anxiety, and stress levels.

Material and method

This cross-sectional study was performed on Taibah University students in Medina from both health-related and non-health-related colleges. We used a self-administrated questionnaire composed of four sections: the informed consent section; sociodemographic information; the Depression, Anxiety, and Stress Scale (DASS-21), which assessed the depression, anxiety, and stress levels; and a caffeine-measuring questionnaire, which reported daily caffeine intake in milligrams per day.

Result

This cross-sectional study examined a 520 convenience sample of Taibah University students with an age range from 17 to 29 years. The majority of the participants were single (95.2%), most of them were female (73.8%), and slightly more than half (51.5%) were recruited from health-related colleges. According to the study's DASS-21 score results, 45.8% of the students had extremely severe stress, 61% had extremely severe anxiety, and 51% had extremely severe depression. The most frequently reported sources of daily caffeine among the participants were Arabic coffee (69.6%), specialty coffee (57.5%), black tea (56.3%), cola (48.7%), and regular coffee (48.5%). The overall daily amount of consumed caffeine ranged from zero to 4276.7 mg/oz. However, no significant association was found between the severity of the DASS-21 score and the daily consumption of caffeine among Taibah University students.

Conclusion

Our study shows no significant association between the severity of depression, anxiety, and stress and daily caffeine consumption among university students. This proves the opposite of the theory that high levels of caffeine consumption can be correlated to high levels of depression, stress, and anxiety.

## Introduction

Caffeine is a central nervous system stimulant that is most commonly found in natural products such as coffee beans, cacao beans, and some types of tea. Caffeine is also added to other products such as energy drinks, soft drinks, and some over-the-counter medications [1]. Coffee is the main vessel for the daily caffeine intake in adults [2]. With its increasing consumption, caffeine has become the most widely used psychoactive stimulant worldwide [1,3]. Motivations for caffeine intake include improving mood, alertness, concentration, and physical performance [4]. Consumption of caffeine has been shown to decrease the risk of obesity, type 2 Diabetes Mellitus, and metabolic syndrome, and it has a significant effect in the prevention of neurodegenerative diseases such as Alzheimer's and Parkinson's disease [3,5].

The recommended daily dosage of caffeine intake for adults is 400 mg (1-4 cups per day), according to the European Food Safety Authority [6]. However, consumption of more than the recommended dosage can cause caffeine intoxication, also known as caffeinism [7]. This manifests as agitation, insomnia, and gastrointestinal symptoms [7]. The average caffeine intake is around 180 mg/day in the general adult population, while college students consume around 268 mg/day [8,9].

A high prevalence of mental health problems, including depression, anxiety, and stress, has been found among college students in general. A study conducted at Taibah University showed similar results for medical students [10]. Other studies conducted on the general population have shown that consumption of the recommended dosage of caffeine was associated with a decreased risk of depression but an increase in anxiety symptoms [11,12].

Due to these results and the lack of studies assessing the correlation between caffeine consumption and depression, anxiety, and stress levels among Taibah University students, our study aims to collect data and evaluate the association between the amount of caffeine consumed by Taibah University students and their levels of depression, anxiety, and stress.

## Materials and methods

Study population and sample size

This cross-sectional study examined a convenience sample of students, all of whom were Arabic speakers, 18 years of age or above, and attending health-related or non-health-related colleges at Taibah University, Medina, Saudi Arabia. Taibah University is one of the largest universities in the western region of Saudi Arabia, with six branches and sixteen colleges that accommodate a total of 69,210 students during the academic year 2022. The inclusion criteria for the study were that participants must be Taibah University students living in Medina, and the exclusion criteria omitted postgraduate students.

Based on the findings from previous literature [13], the estimated means for depression, anxiety, and stress were determined to be 14.5233, 14.21, and 16.0367, respectively, with corresponding standard errors of 9.0133, 9.0767, and 9.2633. To conduct a statistically meaningful study with a high level of confidence, a sample size calculation was performed using G*Power software (Dusseldorf University, Germany). In this calculation, an alpha (significance level) of 0.05, corresponding to a confidence level of 95%, was chosen. Additionally, a beta of 0.20, indicating a desired study power of 80%, was selected as a parameter. The outcome of this sample size calculation suggests that a minimum of 421 participants would be required to conduct the study.

A self-administered online questionnaire was distributed in July 2022 to 69,210 students, of which 520 responded and agreed to participate. The rest didn't respond to the message neither refusing nor agreeing to participate. Of 520 students, none of them withdrew from the study. All participants were asked to fill in the four parts of the questionnaire, and they were informed of their right to not participate at all and that it would have no negative impact on their academic status. Additionally, they were informed of their right to withdraw at any time, and they were assured that there were no right or wrong answers.

Data collection tool

The study used a structured, validated, self-administrated online questionnaire in Arabic that was designed with Google Forms and distributed to the participants via social media (WhatsApp groups and Twitter). The questionnaire is divided into four sections based on close-ended questions. The first section requested informed consent. The second section consisted of a set of questions regarding participants' sociodemographic information (gender, age, material status, college, smoking history, recent diagnosis or history of major depressive disorder and/or anxiety disorders). The third section contained an Arabic version of the Depression, Anxiety, and Stress Scale (DASS-21), which was published in 2016 by Taouk et. al research and cited by more than 100 researchers [14]. 

The DASS-21 is a set of three self-report scales designed to measure the negative emotional states of depression, anxiety, and stress. Each of the three DASS-21 scales contains seven items that are further divided into subscales of two to five items. The depression scale assesses dysphoria, hopelessness, devaluation of life, self-deprecation, lack of interest/involvement in activities, anhedonia, and inertia. The anxiety scale addresses autonomic arousal, skeletal muscle effects, situational anxiety, and subjective experience of anxious affect. The stress scale evaluates factors relating to levels of chronic non-specific arousal: difficulty relaxing and nervous arousal as well as being easily upset/agitated, irritable/over-reactive, and impatient. Participants were asked to use four-point severity/frequency scales to rate the extent to which they experienced each state over the previous week. Scores for depression, anxiety, and stress were calculated by summing the scores for the relevant items.

The fourth section focused on measuring daily caffeine consumption with a valid and reliable scale that had been used in a previous study in Riyadh to assess caffeine consumption and caffeine intoxication [15]. This section investigated various commonly consumed caffeinated drinks, including coffee, decaffeinated coffee, tea, cola, citrus beverages, and energy drinks. An accompanying reference image was provided to show the size and fluid ounces of each drink. Participants were requested to indicate the size and quantity of these drinks they regularly consumed. The amount of caffeine in each drink size was calculated and multiplied by the corresponding daily consumption in cups or cans. These individual caffeine amounts were then summed to determine the total milligrams of caffeine consumed per day. Participants' caffeine intake was categorized as either "low" (less than 400 mg per day) or "high" (more than 400 mg per day), according to the United States Food and Drug Administration (FDA) recommendation for daily caffeine intake [16]. Furthermore, the questionnaire was initially piloted on 15 participants and was proven effective and easy to apply.

Ethical considerations

This study was ethically approved by the Research Ethical Committee of Taibah University in Madinah, Saudi Arabia, study ID TU-006-22. (Reference Number: IORG0008716 - IRB00010413). Personal identification information was not collected, ensuring that the records remained confidential. Informed consent, in electronic form, was obtained from the students after explaining the research purpose to them. The collected data was exclusively used for research purposes.

Data analysis

Statistical analysis was performed using the SPSS 26.0 software package (IBM Corp., Armonk, USA). Descriptive statistics were performed using frequency and percentages for categorical variables, mean range, and standard deviation (SD) for continuous numerical variables. A test of normality (Shapiro-Wilk test) was performed to determine the variable of daily caffeine consumption. Since this was abnormally distributed, it was described using the median and interquartile range (IQR), and non-parametric statistical tests were adopted for statistical analysis (Mann-Whitney, Kruskal-Wallis, and Spearman's tests). Statistical significance was determined at p<0.05 throughout the study.

## Results

Demographic and medical characteristics

**Figure 1:**
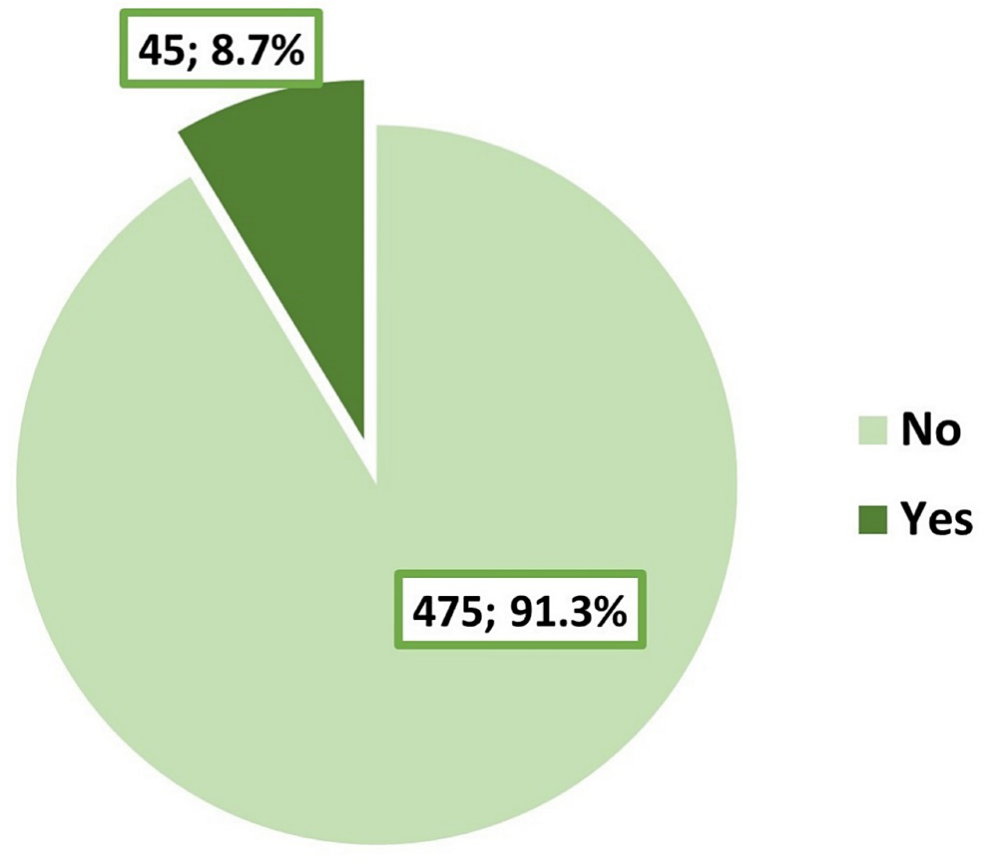
Smoking status of the participating students of Taibah University, Medina (n=520)

**Figure 2:**
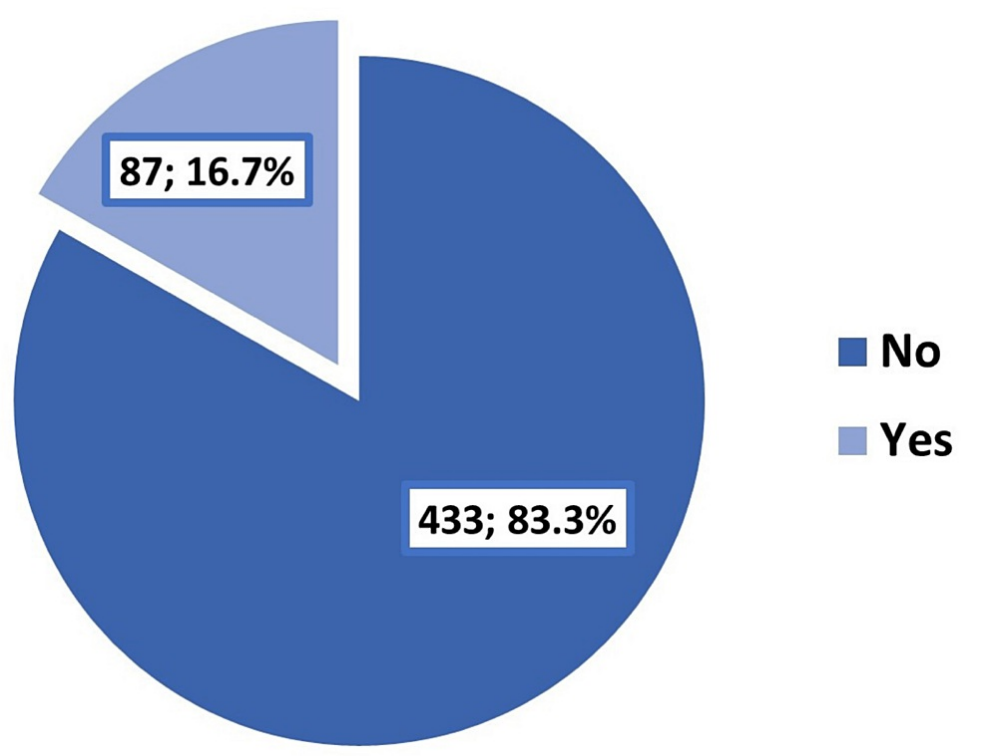
History of depression/anxiety disorders diagnosed by psychiatrists among the participating students of Taibah University, Medina (n=520)

A total of 520 students were included in the study. The demographic characteristics of the participants are summarized in Table 1. The participants ranged in age from 17 to 29 years, with an arithmetic mean of 21.4 years and a standard deviation of 1.9 years. Most of them (73.8%) were female, and the majority were single/unmarried (95.2%). Slightly more than half of the participants (51.5%) were recruited from medical colleges. Smoking was reported by 8.7% of the students, as illustrated in Figure 1. A history of depression/anxiety disorders diagnosed by psychiatrists was reported by 16.7% of the students, as shown in Figure 2.

**Table 1:** Demographic characteristics of the participating students of Taibah University, Medina (n=520) SD: Standard deviation

Variable	Frequency	Percentage
Gender		
Male	136	26.2
Female	384	73.8
Age (years)		
Range	17–29	
Mean±SD	21.4±1.9	
Marital status		
Single	495	95.2
Married	23	4.4
Divorced	2	0.4
College		
Health-Related	268	51.5
Non-Health-Related	252	48.5

Prevalence and severity of depression, stress, and anxiety

**Figure 3:**
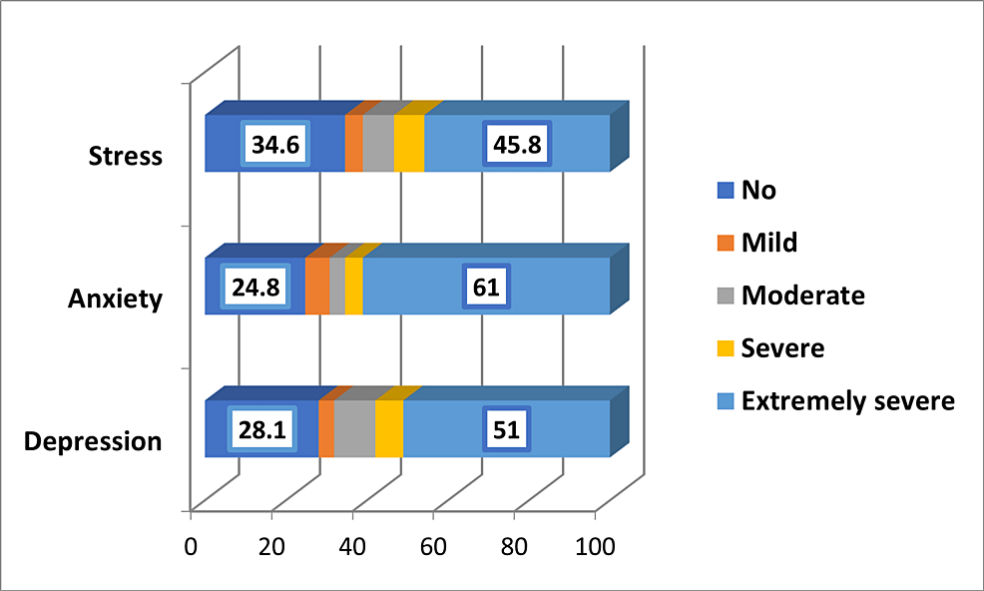
Prevalence of depression, stress, and anxiety among the participants

In Figure 3, it is shown that the prevalence rates of stress, anxiety, and depression among the participants were 65.4%, 75.2%, and 71.9%, respectively. Regarding severity, extremely severe stress, anxiety, and depression were observed in 45.8%, 61%, and 51% of the participants, respectively.

Daily consumption of caffeine among the participants

**Figure 4:**
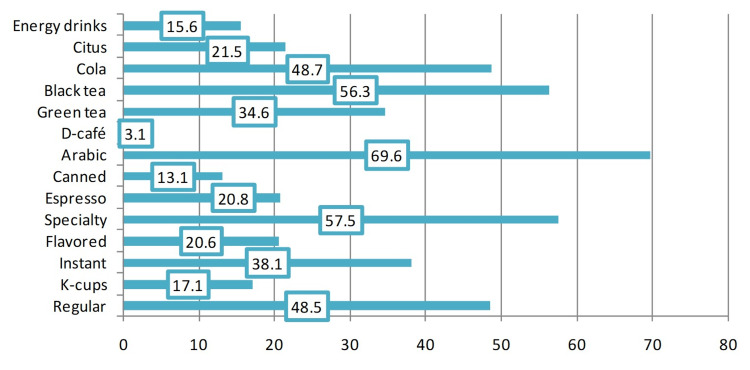
Percentage of daily consumption of caffeine among the participants

**Figure 5:**
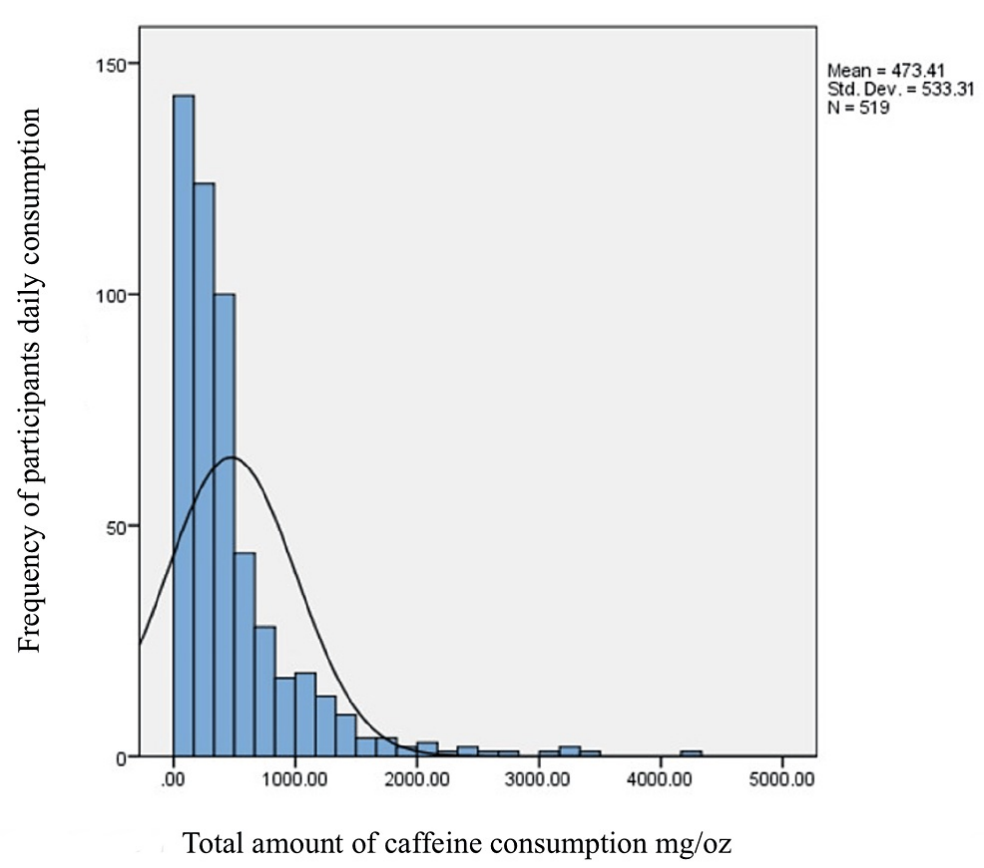
The total amount of daily consumption of caffeine in mg/day among the participants Std. Dev: Standard Deviation; N: number of participants

Figure 4 illustrates that the most frequently reported sources of daily caffeine among the participants were Arabic coffee (69.6%), specialty coffee (57.5%), black tea (56.3%), cola (48.7%), and regular coffee (48.5%). The overall daily amount of consumed caffeine ranged between zero and 4276.7 mg/day. This was abnormally distributed as evidenced by a significant Shapiro-Wilk test (p<0.001), which had a median value of 324.9 mg/day and an interquartile range (IQR) of 155.5-561.7 mg/day (Figure 5).

Factors associated with daily caffeine consumption

Socio-demographic and Medical Factors

Male participants were more likely to consume caffeine compared to female participants (mean ranks were 287.92 and 250.08, respectively; p=0.011). Participants' marital status, age, college, smoking status, and history of depression/anxiety disorders diagnosed by psychiatrists were not significantly associated with daily consumption of caffeine (Table 2).

**Table 2:** Socio-demographic and medical factors associated with daily consumption of caffeine among the participants *Mann-Whitney test; **Kruskal-Wallis test; ˚Spearman`s coefficient of correlation

Variable	Daily consumption of caffeine			p-value
	Median	IQR	Mean rank	
Gender				0.011*
Male	386.6	188.1-92.6	287.92	
Female	298.2	138.5-33.9	250.08	
Age in years	r˚=0.008			0.849˚
Marital status				0.127**
Single	325.8	158-565.7	261.17	
Married	174	127-508.3	220.26	
Divorced	1616.5	445.1-1616.5	427	
College				0.649*
Health-Related colleges	328.7	146.7-536.2	257.10	
Non-Health-Related colleges	321.7	160.5-584.1	263.10	
Smoking				0.539*
No	326.2	155.5-572	261.23	
Yes	275.7	156.2-466.9	246.72	
depression/anxiety disorders diagnosed by psychiatrists				0.251*
No	319.1	155.1-543.4	256.61	
Yes	356.4	155.5-804.4	276.85	

Depression

There was no significant association between the severity of depression and daily consumption of caffeine, as seen in Table 3.

**Table 3:** Association between severity of depression and daily consumption of caffeine among the participants *Kruskal-Wallis test

Severity of depression	Daily consumption of caffeine			p-value*
	Median	IQR	Mean rank	
None	340.2	152.3-720.2	271.48	0.824
Mild	286.9	125.2-525.4	250.25	
Moderate	378.3	128.8-558.4	258.67	
Severe	283.6	170.3-448.3	242.94	
Extremely severe	321.3	156.9-535.7	256.99	

Anxiety

Table 4 shows that there was no significant association between the severity of anxiety and daily consumption of caffeine among the participants.

**Table 4:** Association between severity of anxiety and daily consumption of caffeine among the participants *Kruskal-Wallis test

Severity of anxiety	Daily consumption of caffeine			p-value*
	Median	IQR	Mean rank	
None	339.1	155.3-743.9	272.37	0.557
Mild	387	164-652.7	278.27	
Moderate	241.3	87.5-609.9	237.98	
Severe	290	75.8-459.6	227.09	
Extremely severe	321.5	158.4-525	256.95	

Stress

Stress severity was not significantly associated with daily consumption of caffeine among the participants, as shown in Table 5.

**Table 5:** Association between severity of stress and daily consumption of caffeine among the participants *Kruskal-Wallis test

Severity of stress	Daily consumption of caffeine			p-value*
	Median	IQR	Mean rank	
None	336.7	155.2-696.1	269.56	0.698
Mild	290	75.8-459.6	227.09	
Moderate	326.9	176.2-456.6	254.51	
Severe	310.2	131.4-572.8	246.73	
Extremely severe	325.1	159.6-531.8	259.02	

## Discussion

In this comprehensive cross-sectional study conducted at Taibah University, the Depression Anxiety Stress Scale (DASS-21) was used to examine the association between daily caffeine intake and psychological well-being, including depression, anxiety, and stress, in students from both health-related and non-health-related colleges of Taibah University. Surprisingly, based on the 520 responses, we found that the average caffeine consumption was 473.41 ± 533.31 mg/day, which surpasses the United States Food and Drug Administration's daily caffeine intake limit of 400 mg for adults [16].

This research demonstrated that the prevalence rates of stress, anxiety, and depression among the participants were 65.4%, 75.2%, and 71.9%, respectively. Regarding severity, extremely severe stress, anxiety, and depression were observed in 45.8%, 61%, and 51% of the participants, respectively. However, the analysis confirmed that there was no significant association between the severity of depression, anxiety, or stress and daily consumption of caffeine.

Our results showed an unexpected contrast when compared to a study undertaken at Princess Nourah Bint Abdulrahman University. In this study, there was a clear positive correlation (p<0.045) between the levels of caffeine consumption, intoxication, and their relation to stress was observed [14]. An even more significant difference is evident in the percentage of participants reporting stress levels, with 69.9% indicating moderate stress and 18.7% indicating high stress [15].

Further complicating this relation, research conducted by both Luebbe et al. and James et al. supports our findings, suggesting that there is no significant relationship between caffeine intake and conditions such as depression or anxiety [17,18]. On the other hand, a study conducted by Bertasi et al. among college students found that symptoms of depression, such as poor appetite or overeating, sleep disturbances, and feelings of hopelessness, were positively associated with caffeine consumption [11]. Likewise, a study conducted by Kaplan et al. showed a high risk of depression among the participants who consumed high levels of caffeine (500 mg or more), which opposes the results of the present study [19]. In addition, an analysis of a data survey conducted in 2015 among secondary school children found that the incidence of depression decreased as the level of caffeine intake increased [4]. Another study conducted by Ruusunen et al. reported that middle-aged men noticed that coffee intake reduced the risk of depression, but the dose of caffeine had no association [20]. Moreover, Smith et al. also reported that caffeine consumption was associated with a reduced risk of depression compared to non-consumption [21].

Caffeine has been classically considered to induce anxiety at higher doses, typically over 300 mg, but the consumption of caffeine is poorly correlated with anxiety or anxious traits [22,23]. A cohort study of 3323 students aged 11-17 years (48.5% boys, 51.5% girls) found that the effect of caffeine on anxiety was not significant in girls, but in boys, anxiety increased as caffeine intake increased [24]. Another study found that caffeine can have positive effects in some cases. For instance, low doses of the stimulant have been shown to reduce anxiety and elevate mood [25]. Additionally, a study done by R.S. Lazarus found a significant positive relationship between levels of caffeine consumption and levels of perceived stress. The consumption of caffeinated beverages is a known coping strategy among college students for managing stressful academic situations [26]. However, a study conducted by Gilliland and Andress reported negative effects of caffeine on stress and mental health. The study reported higher anxiety levels in consumers of moderate and high amounts of caffeine compared to those who did not consume caffeine in a student sample [27]. The discrepancies in these statistical findings emphasize the complexity and highly context-dependent nature of the relationship between caffeine consumption and mental health.

Our study shows no significant association between the severity of depression, stress, and anxiety and daily consumption of caffeine (p=0.824, p=0.698, p=0.557) among Taibah University students. These results contradict the theory that consuming high levels of caffeine can have a negative effect on depression, stress, and anxiety [4,12].

Limitation and recommendation

Our study had some limitations. First, the study's generalizability was limited by the convenience sample and participation. Second, despite the fact that this study took into account every popular caffeinated beverage, it excluded other potential sources of caffeine such as chocolate and caffeine tablets. In addition, varying sizes of drinks and relative caffeine concentrations were not considered, which could be corrected by including all sources of caffeine. Additionally, the causation of correlations cannot be found in a cross-sectional study. As a result, the study investigated the association between the studied factors without determining the start of caffeine consumption or the beginning of the symptoms. Another issue is the bias in self-reporting. Finally, the study was carried out after the COVID-19 pandemic, which could have an impact on the findings.

## Conclusions

Taibah University students were studied to identify the relationship between high caffeine intake and DASS-21 scores, but no significant relationship was found. However, high DASS-21 scores were observed among the participants.

Taibah University students must be given significant support and education on common psychological issues from academic advisors. We suggest identifying high-risk students and making psychological assistance easily accessible to address depression, anxiety, and stress among Taibah University students.
